# Clinical application of robotic orthopedic surgery: a bibliometric study

**DOI:** 10.1186/s12891-021-04714-7

**Published:** 2021-11-22

**Authors:** Cheng Li, Lei Wang, Carsten Perka, Andrej Trampuz

**Affiliations:** 1 Department of Orthopaedic Surgery, Beijing Jishuitan Hospital, Fourth Clinical College of Peking University, Beijing, P.R. China; 2 Center for Musculoskeletal Surgery (CMSC), Charité – Universitätsmedizin Berlin, corporate member of Freie Universität Berlin, Humboldt-Universität zu Berlin, and Berlin Institute of Health, Berlin, Germany

**Keywords:** Bibliometrics, Research, Robotic surgical procedures, Orthopedics

## Abstract

**Objectives:**

The present study aimed to evaluate the status and trends of robotic orthopedic surgery in a clinical setting using bibliometrics.

**Methods:**

All relevant publications on the clinical use of robotic surgery in orthopedics were searched from the Web of Science database. Subsequently, data were analyzed using bibliometrics. Visualizing data of bibliographic coupling, co-citation, and co-occurrence analysis were performed using VOSviewer.

**Results:**

In total, 224 clinical studies met the included standards between 2000 to 2019. Global publications presented an increasing annual trend, with the United States found to have the largest number of publications and robotic companies active in the field (*n* = 99), followed by China (*n* = 38), and the United Kingdom (*n* = 27). The institution with the most contributions was the Beijing Jishuitan Hospital in China (*n* = 15). The most productive scholars were Tian Wei and Mont Michael A, with 14 publications each. The top 30 most cited papers list showed 29 publications to be cited on more than 40 occassions. The journal with the most related and influential publications on robotic orthopedic surgery was the Journal of Arthroplasty. Fourteen types of robots were used, with the majority applied in knee and spinal surgery. MAKO was the most widely used robot in hip and knee surgery and Mazor in spinal surgery. Most studies were small sample populations of low-quality in this field. The top 20 most frequently used keywords were identified from 950 author keywords. Research on orthopedic robots were classified into two clusters by co-occurrence networks: spinal-related robotic surgery and joint-related robotic surgery.

**Conclusions:**

The present bibliometric study summarizes the clinical research of orthopedic robots on study type, sample size, type of surgery, robot information, surgical site, most popular keywords, most cited papers, journals, authors, institutions, and countries. These findings may assist the scholars better understand the current status and research trends to guide future practice and directions.

**Supplementary Information:**

The online version contains supplementary material available at 10.1186/s12891-021-04714-7.

## Introduction

Orthopedic techniques are rapidly expanding, particularly in the technological revolution of deep learning, robotic surgery, virtual reality, and 3D printing [1–4]. Orthopedic robotics is a complex as well as extremely challenging technique and is involved in multidisciplinary research from design to clinical use. Nonetheless, robotic techniques have promising applications in orthopedic surgery. In recent years, robotic technology has matured gradually, with an increasing number of surgical robots obtaining approval from the Food and Drug Administration for clinical practice [5–8]. Some reports found that robotic surgery is more accurate in orthopedic implant placement, results in less intraoperative radiation exposure, as well as postoperative bleeding and pain, and has a better prognosis compared with conventional freehand techniques [9–12]. Meanwhile, some influential factors may prevent the development of orthopedic robotics. For the hospitals of underdeveloped and developing countries, the financial burden of purchase and maintenance costs most likely limits its development [13–15]. Furthermore, it poses a challenge for orthopedic surgeons to change the conventional concept and skills in robotic techniques [15, 16]. The development of the clinical application of orthopedic robotic techniques under these multiple uncertainty factors remains unknown. To help researchers gain insight into robotic surgery in orthopedics, discovering the current developmental status and hot spots of robotic technique in different orthopedic subspecialties is required.

Bibliometrics is a research method used to provide the characteristics and development of a subject area [17–20]. It is usually combined with visualization information to find the relationship between institutions, journals, and countries, and identify research trends [21, 22]. To our knowledge, there is no research on a fully comprehensive assessment of the clinical research of orthopedic robots by bibliometric analysis.

The objective of this study was: a) using bibliometric analysis to discover the features in robotic orthopedic surgery from study design, type of surgery, robot information, surgical site, most popular keywords, most cited papers, journals, authors, institutions, and countries; b) data visualization to reflect the relationship between different institutions, journals, countries and explore research hotspots and trends.

## Methods

### Search strategy

The electronic database of Web of Science (WoS; SCI-Expanded) was searched between 2000 and 2019 without language restrictions, with the following search strategy applied: “arthroplasty OR joint replacement OR spinal surgery OR spine surgery OR trauma OR orthopaedics OR orthopaedic OR orthopedics OR orthopedic” and “robotic surgery OR robotic technology OR robotic systems OR robotics OR robot-assisted surgery OR robot assisted surgery OR robotic-arm assisted surgery OR robotic arm assisted surgery”. Cited literature from search results were also reviewed to supplement articles not found by the search strategy.

### Inclusion and exclusion criteria

Inclusion criteria for further analysis were based on the following: (a) article describing a clinical study on the application of robotic surgery in orthopedics; and (b) review article, meta-analysis, clinical trial, and guideline. Exclusion criteria were book chapters, conference proceedings, animal studies, and cadaveric investigations.

### Data extraction and visualization

The following information were exported from the WoS in a text file format and further summarized and analyzed using WPS office (Version 11.2.0.9232; Kingsoft): author, article type, citations, country, digital object identifier, impact factor, journal, institution, keywords, sample size, study type, title, and year of publication.

All records of the WoS database were exported as a text format and further imported into VOSviewer (version 1.6.13; Leiden University), with the frequently used approach of co-citation, coupling, co-occurrence analysis performed in the present study. Co-citation analysis, which occurs when two documents are referenced together in a third document [23]. Coupling analysis is in a static form, which is based on the number of identical references between two documents. Compared with coupling analysis, co-citation analysis is in a dynamic form. Both approaches supplement each other [24]. Therefore, we both methods were performed to demonstrate the relationship among different journals, institutions, and countries. Co-occurrence analysis is calculated by all keywords to identify high-frequency subject terms and research directions [20].

## Results

Among the 1013 primary research results, 186 publications were identified after reviewing the title, abstract, and full-text. From the included studies, 1124 cited papers were reviewed. Finally, 224 clinically-related publications were identified for bibliometric analysis, comprising 164 articles, 13 meta-analyses, and 47 reviews. Two hundred and nineteen were in the English language, whereas the remaining five were in German.

### Countries

**Fig. 1:**
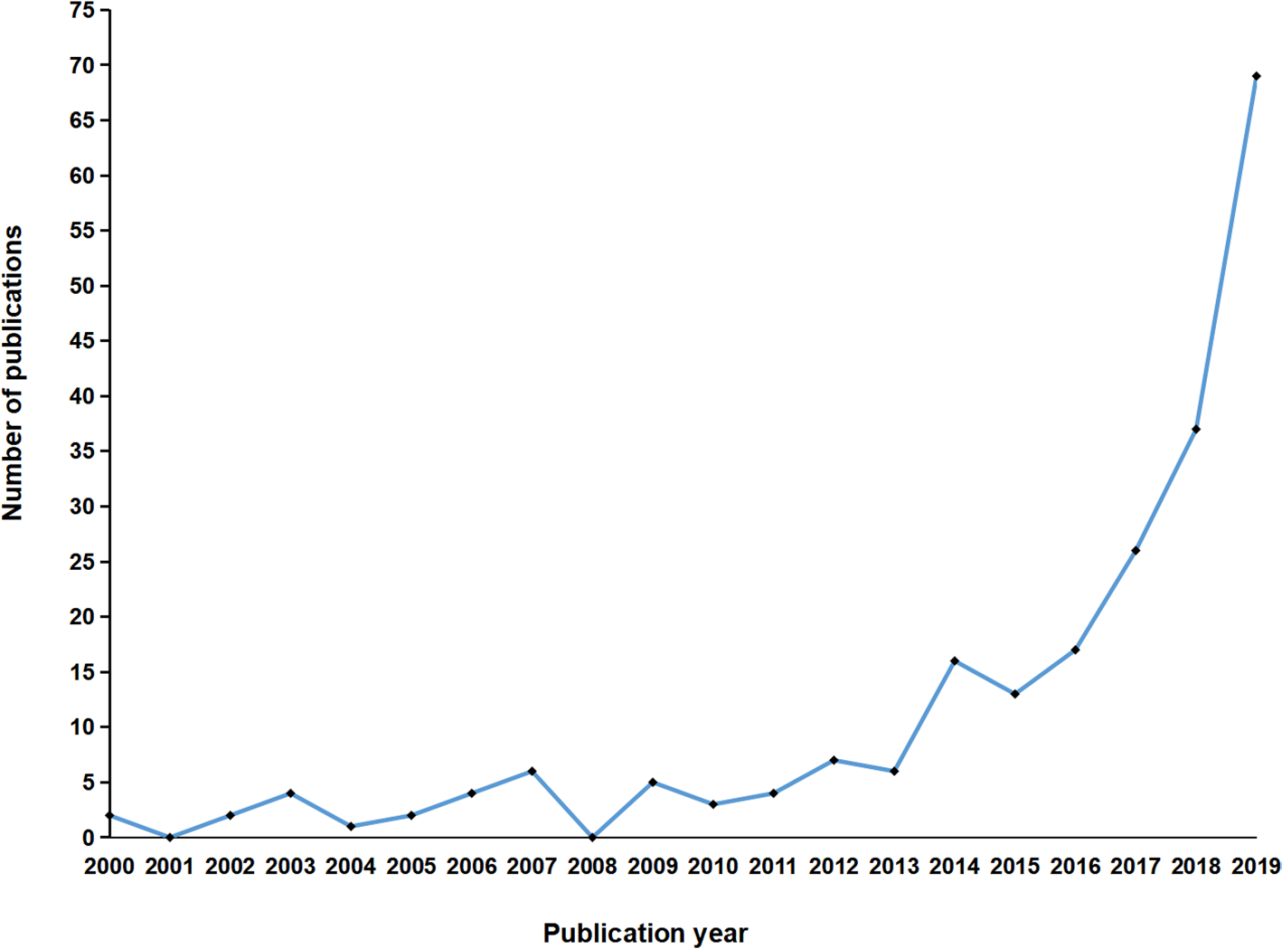
Graphs indicating the total annual number of global contributions

**Fig. 2:**
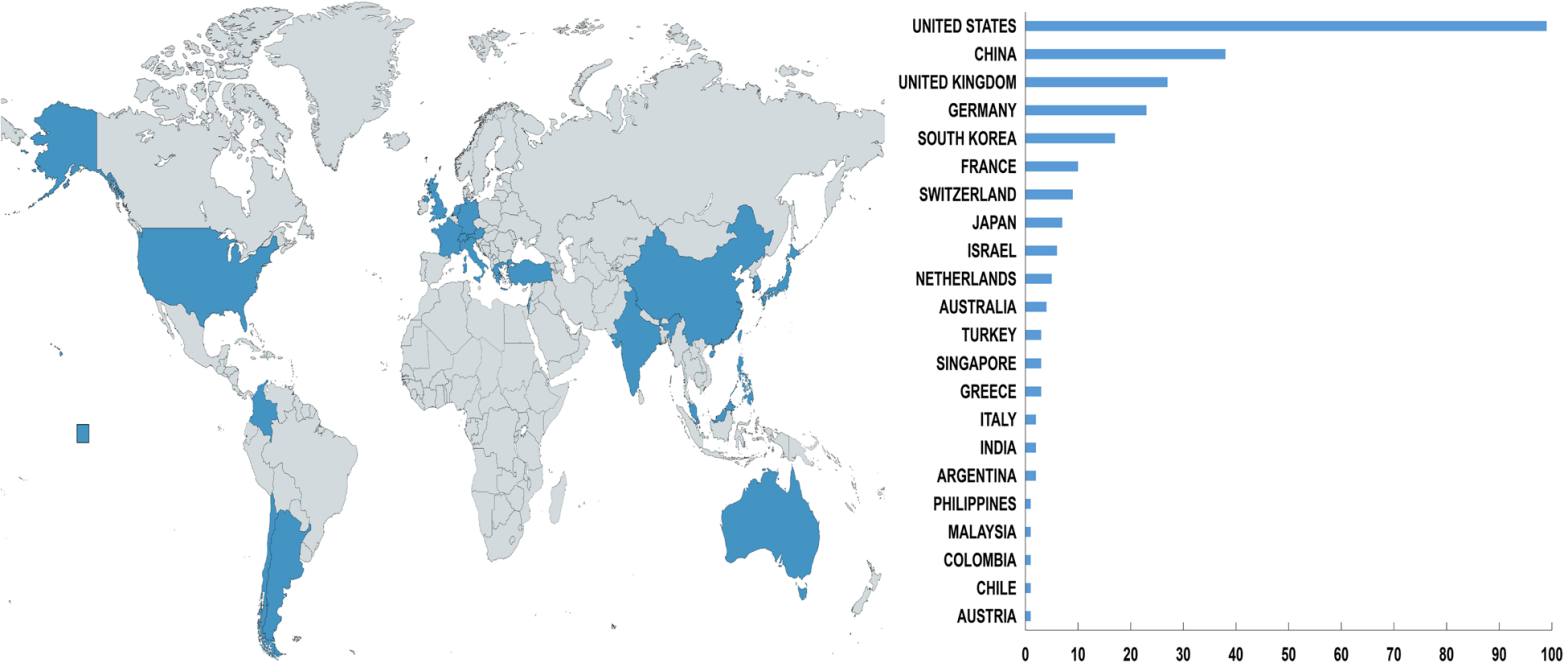
Global distribution according to country

**Fig. 3:**
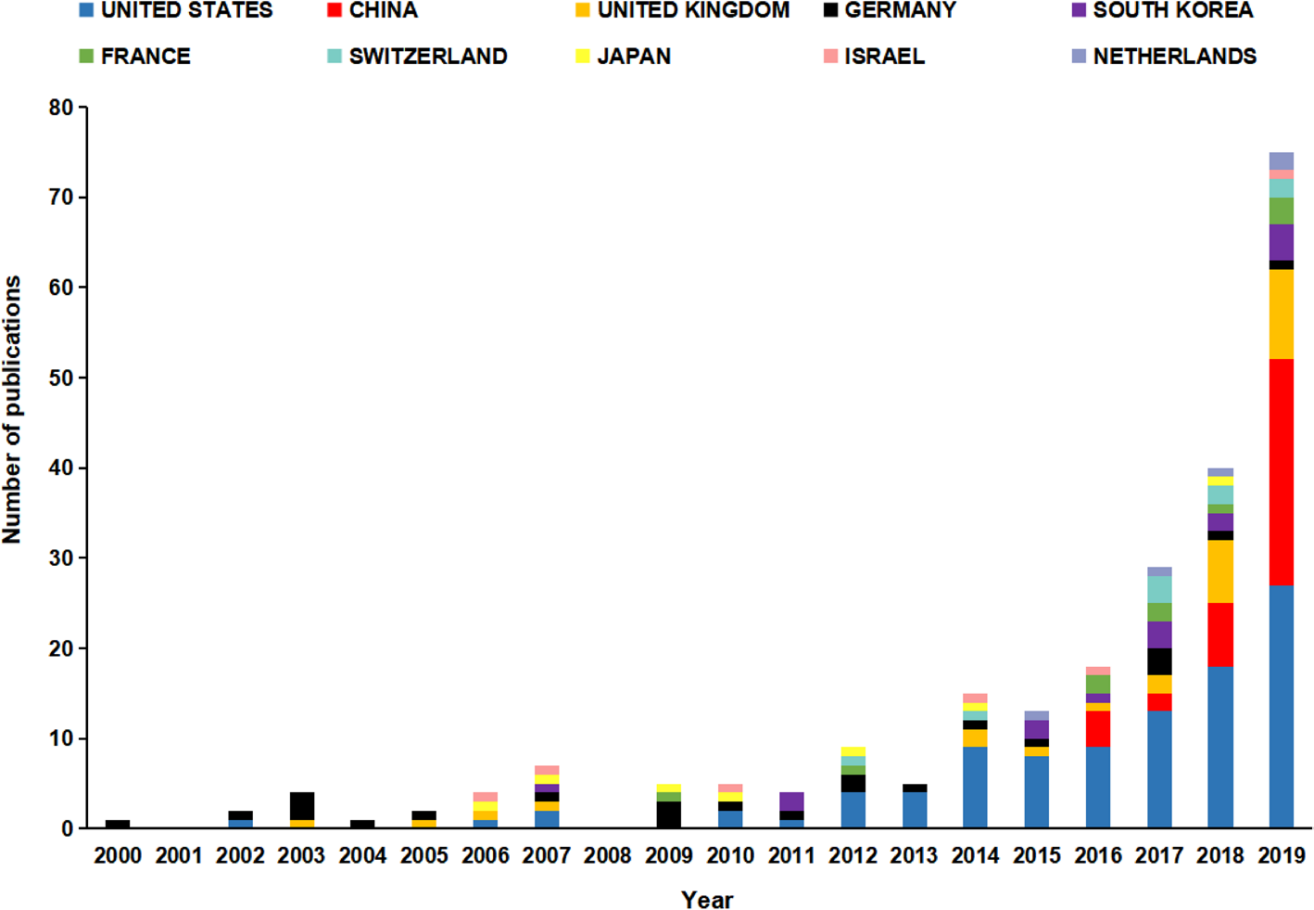
Annual contributions according to country

The overall global contribution of publications appeared in an increasing annual trend from 2000 to 2019 (Fig. 1). Publications originated from 23 countries, with the United States the largest contributor (*n* = 99), followed by China (*n* = 38), the United Kingdom (*n* = 27), Germany (*n* = 23), South Korea (*n* = 17), and France (*n* = 10; Fig. 2). In the years 2018 and 2019, more than 79% (32/40, 62/86, respectively) of global contributions were made by the United States, China, and the United Kingdom (Fig. 3).

**Fig. 4:**
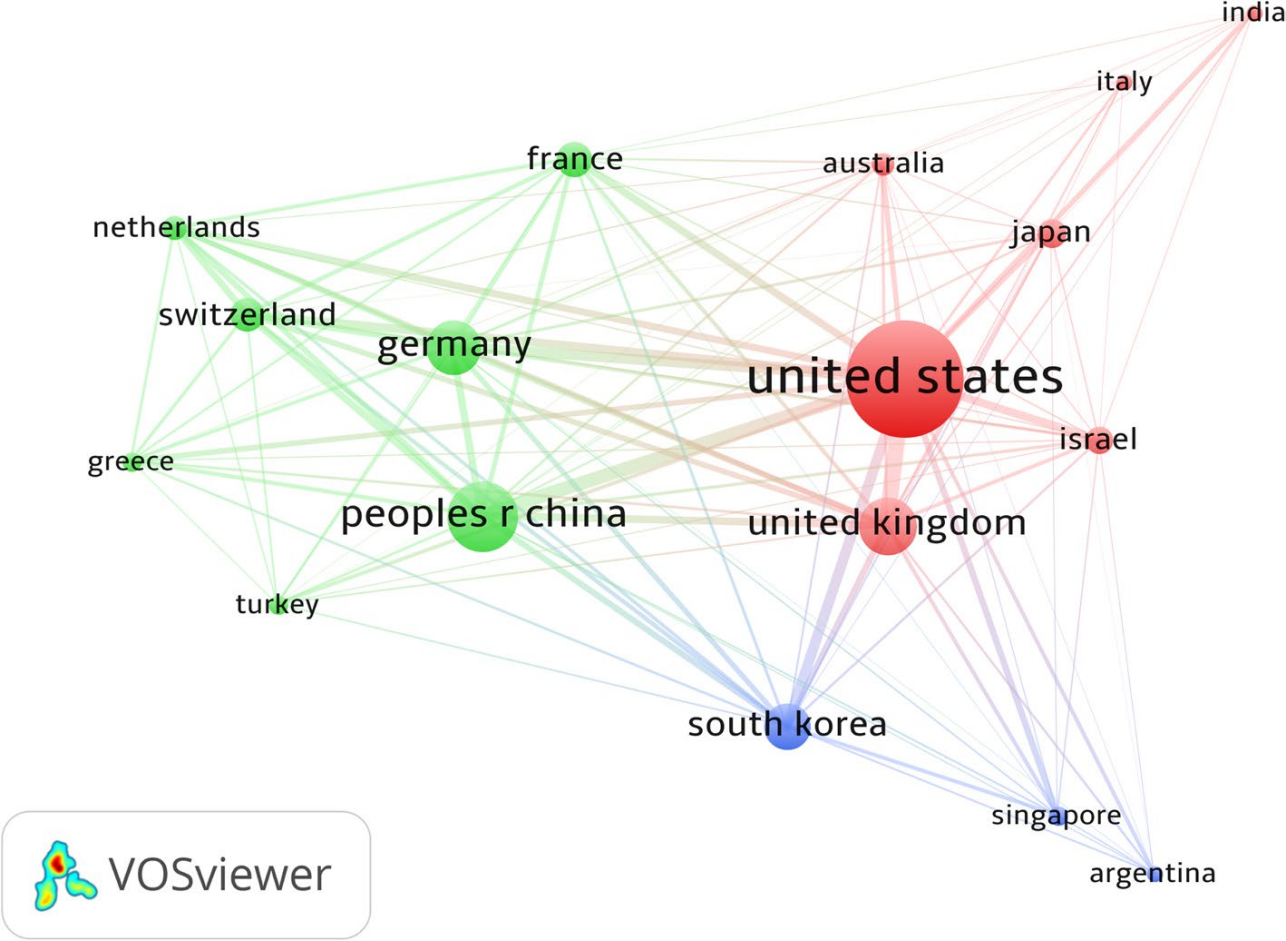
Coupling analysis of countries on global robotic orthopedic surgery research

The minimum number of publications of a country was next set to at least two publications. Seventeen countries met the criteria. The top three countries with the highest total link strengths were the United States (*n* = 13,754), the United Kingdom (*n* = 6 903), and China (*n* = 6 119; Fig. 4).

### Organizations

Six institutions published at least eight publications. The organization with the greatest number of publications was the Beijing Jishuitan Hospital with 15 papers, followed by the Cleveland Clinic, and University of London (13 papers each; Table 1).

**Table 1:** Global institutions with at least eight publications on orthopedic robotic surgery

Institutions	Number of articles	Country	H-index	Sum of Times Cited	Average citations per item
Beijing Jishuitan Hospital	15	China	5	97	6.47
Cleveland Clinic	13	USA	7	145	11.15
University of London	13	UK	7	140	10.77
Hospital for Special Surgery	10	USA	7	178	17.8
Northwell Health	8	USA	4	33	4.13
University of Göttingen	8	Germany	7	404	50.5

**Fig. 5:**
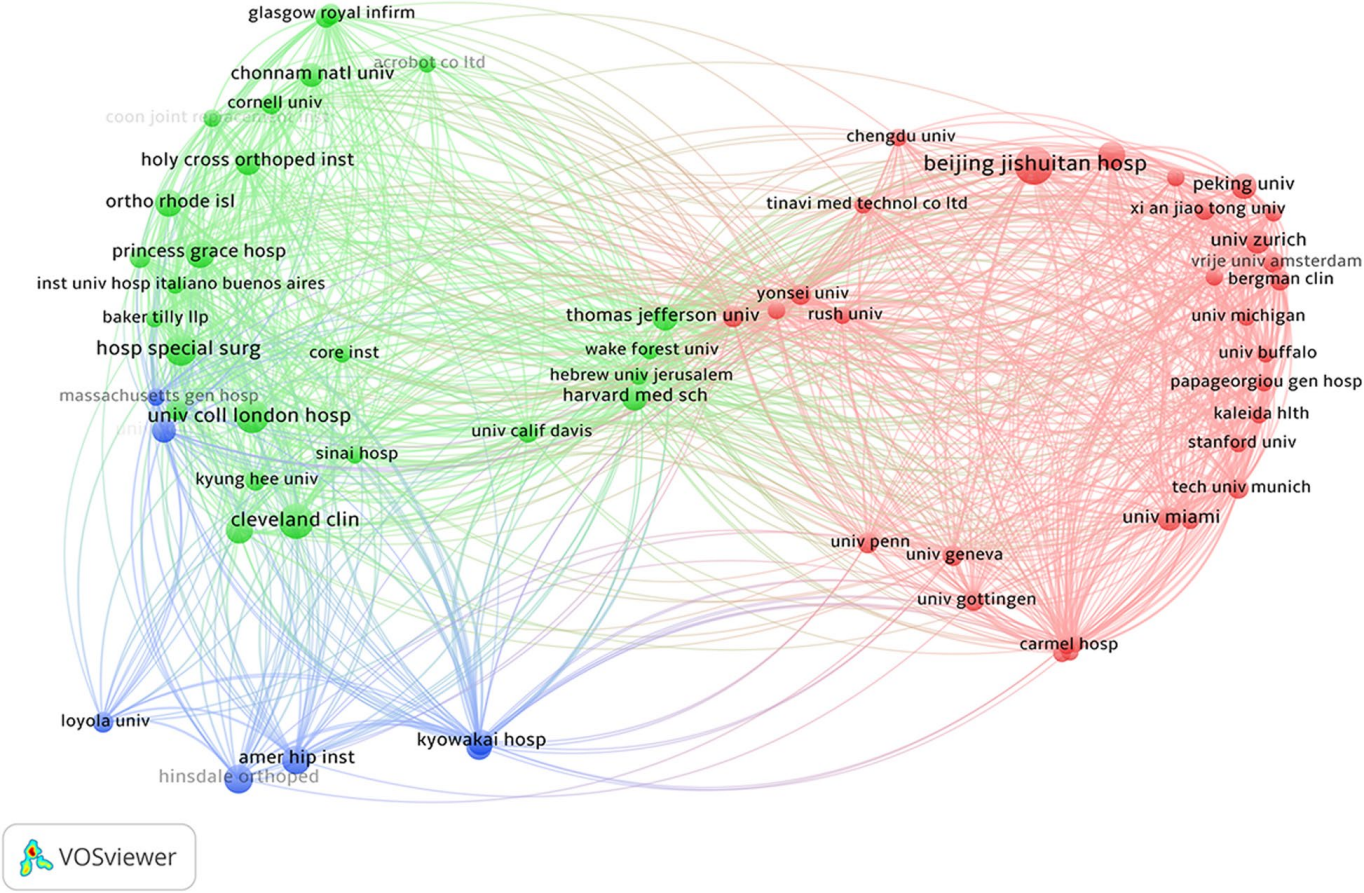
Coupling analysis of institutions on robotic orthopedic surgery

From 63 organizations, coupling analysis (threshold: two papers) showed that the top three institutions with the greatest total link strengths were the Cleveland Clinic (*n* = 2639), followed by Beijing Jishuitan Hospital (*n* = 2483), and University College Hospital (*n* = 2432; Fig. 5).

### Authors

Nine authors contributed at least nine papers. Tian Wei and Mont Michael A had 14 publications, followed by Domb Benjamin G and Liu Ya-Jun with ten papers each, and Haddad Fares S with nine. Mont Michael A had the highest H-index, whereas Domb Benjamin G had the highest number of total and average citations among the top nine authors (Table 2).

**Table 2:** Top nine authors in the orthopedic robotic surgery field ranked according to the number of publications

Author name	Number of article	Country	Institution	h-index	Sum of times cited	Average citations per item
Mont, Michael A	14	USA	Cleveland Clinic	7	115	8.21
Tian, Wei	14	China	Beijing Jishuitan Hospital	5	84	6
Domb, Benjamin G	10	USA	Hinsdale Orthopaedics	6	153	15.3
Liu, Ya-Jun	10	China	Beijing Jishuitan Hospital	5	59	5.9
Haddad, Fares S	9	UK	University of London	5	91	10.11
Khlopas, Anton	9	USA	Cleveland Clinic	6	83	9.22
Konan, Sujith	9	UK	University of London	5	88	9.78
Sodhi, Nipun	9	USA	Cleveland Clinic	6	81	9
Kayani, Babar	9	UK	University of London	5	91	10.11

### Citation and journals

From the top 30 most cited papers on robotic orthopedic surgery, 29 articles were cited on more than 40 occassions (Additional file 1). Of these, *Spine* had the greatest number of publications with five papers. *The Journal of Bone and Joint Surgery—American Volume, Clinical Orthopaedics and Related Research,* and *Journal of Arthroplasty* had four papers each. The publication by J Cobb and colleagues had the highest number of citations (*n* = 153) [25], followed by Devito DP et. al. (*n* = 126) [26], and Kantelhardt SR et. al. (*n* = 119) [11].

The use of robotics in orthopedic surgery was published in 69 journals. The journal with the highest number of published articles was the *Journal of Arthroplasty* (*n* = 27) and was also found to have the highest total times cited (*n* = 238). The next journals with the highest number of articles were *Spine, International Journal of Medical Robotics and Computer Assisted Surgery*, and *Orthopaedic Surgery,* all sharing an equal number of publications (*n* = 13; Table 3). According to the Journal Citation Reports 2018, *Sports Medicine* had the highest impact factor (7.583) among all journals, followed by *The Journal of Bone and Joint Surgery—American Volume* (4.716), and *Neurosurgery* (4.605; Table 4).

**Table 3:** Journals with at least six publications in orthopedic robotic surgery

Journals	Total publications	Total cites	Average citations per item
Journal of Arthroplasty	27	238	8.81
Spine	13	212	16.31
International Journal of Medical Robotics and Computer Assisted Surgery	13	78	6
Orthopaedic Surgery	13	15	1.15
World Neurosurgery	10	26	2.6
Knee Surgery, Sports Traumatology, Arthroscopy	9	62	6.89
Journal of Knee Surgery	9	30	3.33
Neurosurgical Focus	7	48	6.86
Bone & Joint Journal	7	18	2.57
Clinical Orthopaedics and Related Research	6	104	17.33
European Spine Journal	6	108	18
Expert Review of Medical Devices	6	20	3.33
Knee	6	54	9

**Table 4:** Top 10 journals on orthopedic robotic surgery ranked according to impact factor

Journals	Impact factor (2018)	Total publications
Sports Medicine	7.583	1
Journal of Bone and Joint Surgery-American Volume	4.716	4
Neurosurgery	4.605	4
Bone & Joint Journal	4.301	7
Clinical Orthopaedics and Related Research	4.154	6
Scientific Reports	4.011	1
Plastic and Reconstructive Surgery	3.946	1
Annals of Translational Medicine	3.689	3
Bone & Joint Research	3.652	1
Haemophilia	3.59	1

**Fig. 6:**
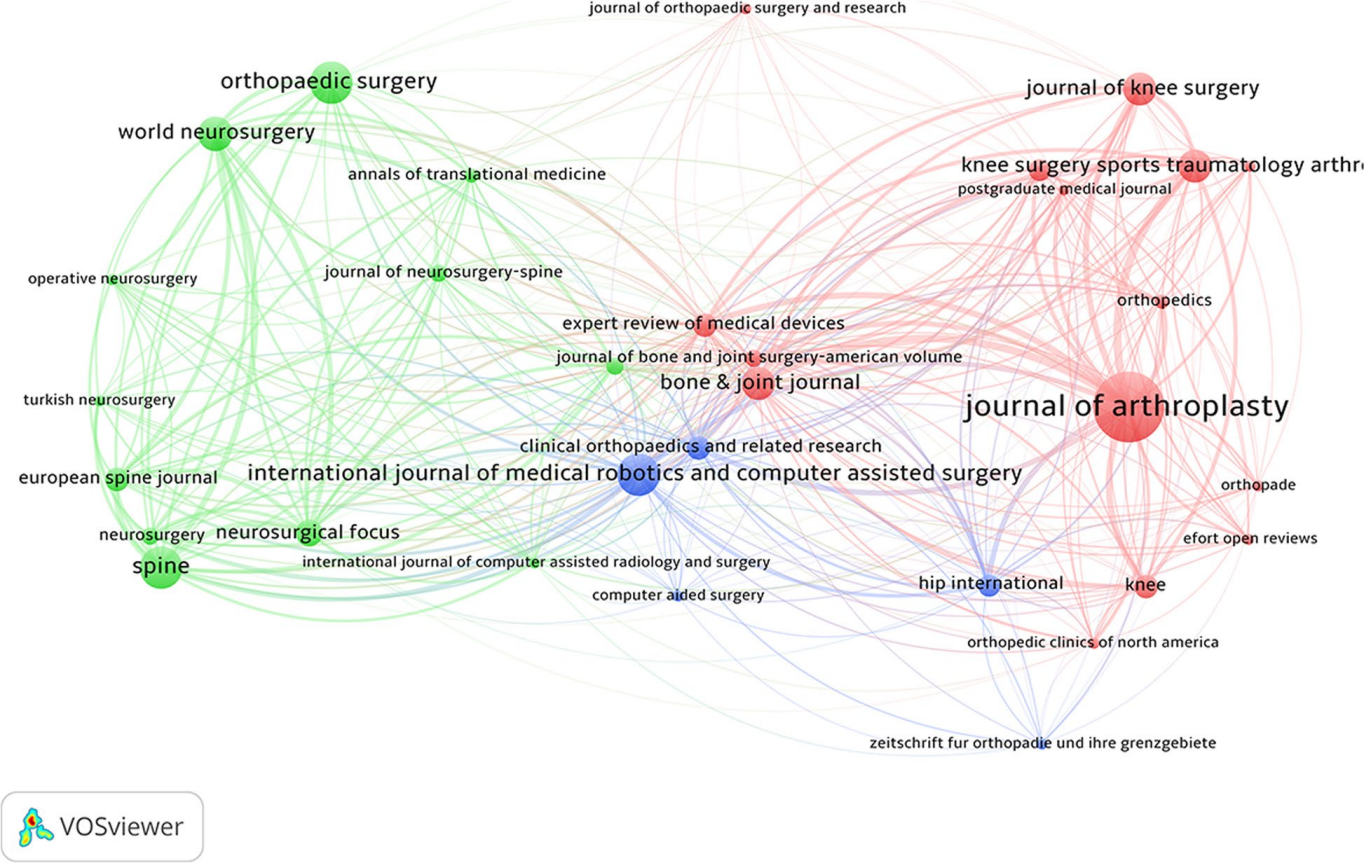
Coupling analysis of journals on robotic orthopedic surgery

**Fig. 7:**
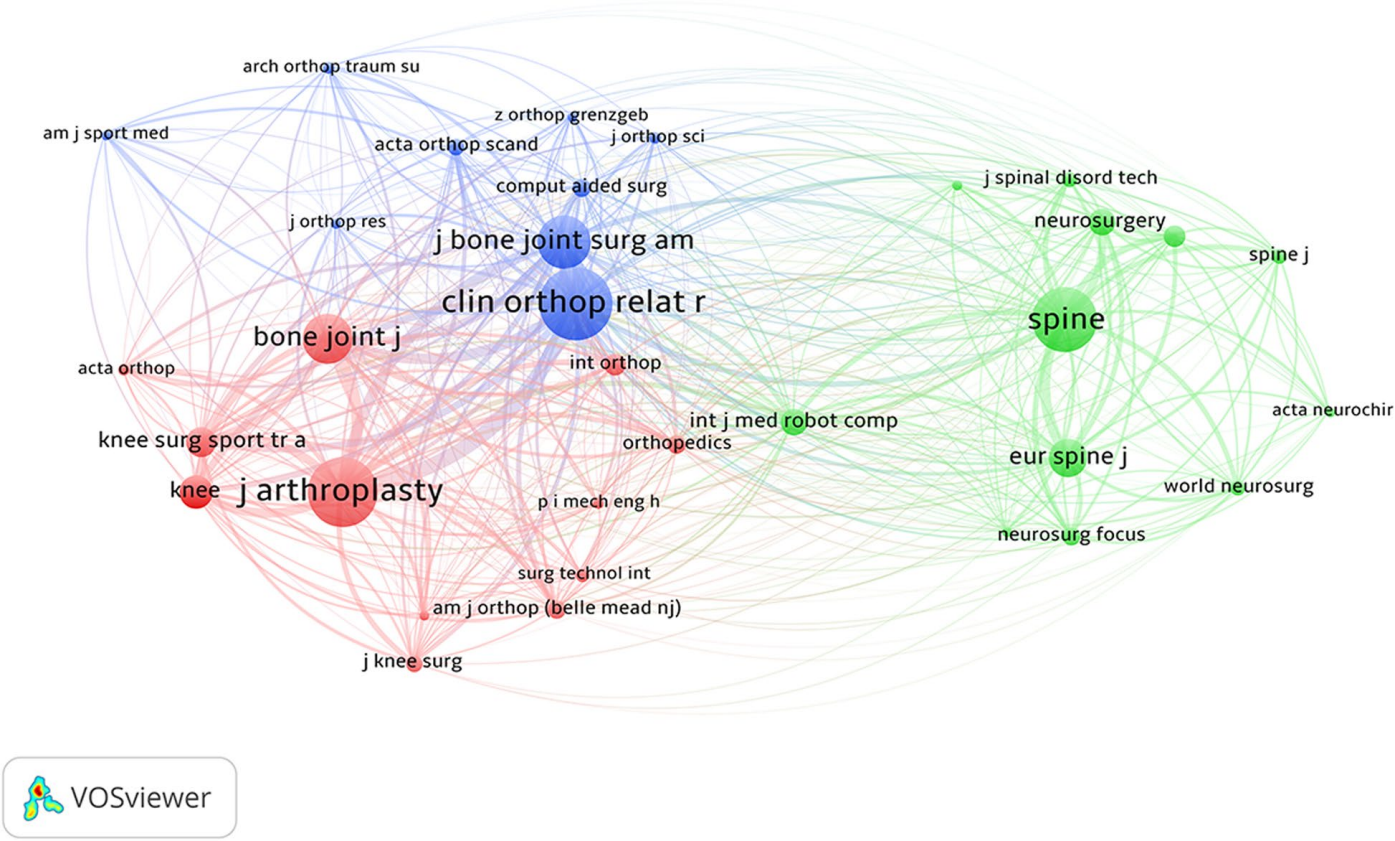
Co-citation analysis of journals on robotic orthopedic surgery

The minimum number of publications of a journal was next set to at least two papers. Coupling analysis of 32 journals was performed, revealing the *Journal of Arthroplasty* (*n* = 3511), *Spine* (*n* = 1914), and *Bone & Joint Journal* (*n* = 1691) to have the greatest total link strengths (Fig. 6). The minimum number of publications of a journal was next set to at least 30 citations. Co-citation analysis of 30 journals was demonstrated that the highest total link strengths in the *Journal of Arthroplasty* (*n* = 20,299), followed by the *Clinical Orthopaedics and Related Research* (*n* = 19,690), and *Journal of Bone and Joint Surgery American Volume* (*n* = 12,359; Fig. 7). The circle size of coupling analysis indicates the number of publications. In contrast, the circle size of co-citation analysis indicates the number of citations. The closer the circle's distance, the more similar the subject. The thickness of the connecting line represents the link strength of the network between the circle (Figs. 6 and 7).

### Type of robotic surgery and location

**Fig. 8:**
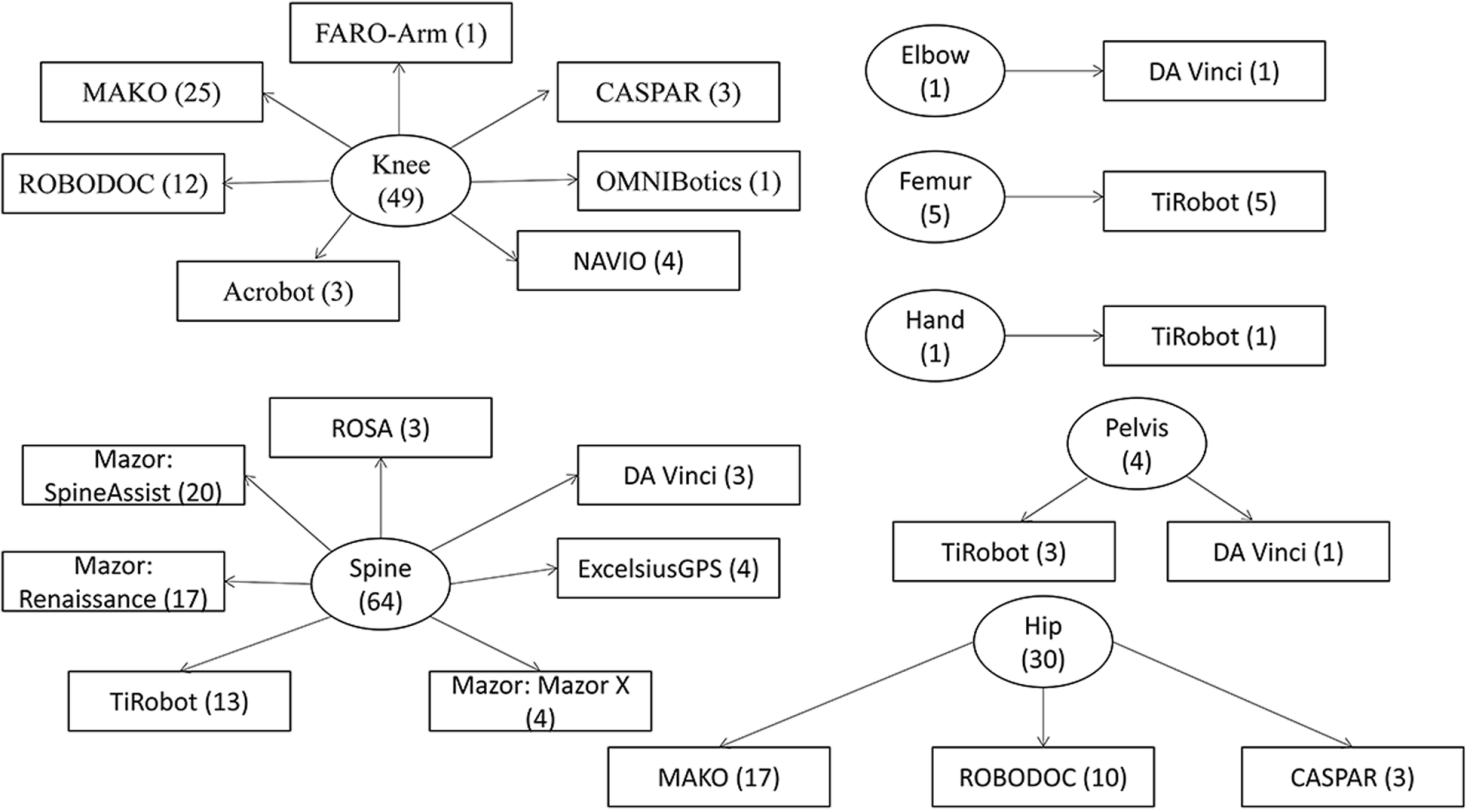
Types of robots in orthopedic surgery and corresponding surgical sites

**Fig. 9:**
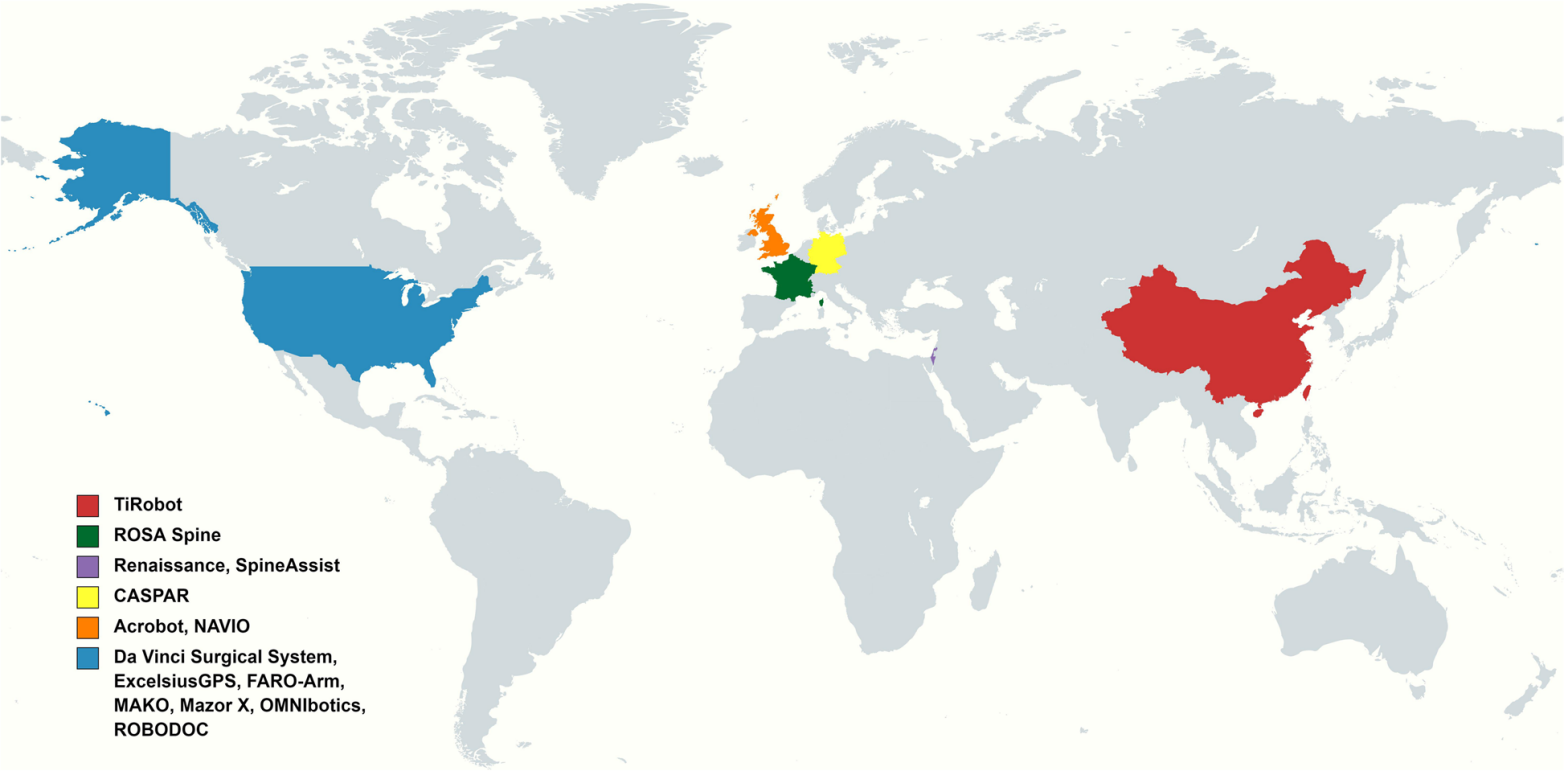
Six countries that produce robots for orthopedic use

After excluding unavailable information, one hundred and fifty-two clinical studies described the type of robot and surgical information. The top three most common surgical sites were the spine, knee, and hip. Pedicle screw implantation was the most performed surgical procedure of the spine, and total joint arthroplasty was most frequently reported in the hip and knee (Table 5). Fourteen types of orthopedic robots were used in seven surgical sites, with the most diverse number of types used in the knee and spine (*n* = 7 types each), followed by the hip (*n* = 3), and pelvis (*n* = 2). TiRobot was the most widely used robot in the different surgical sites (four positions), followed by DA Vinci (three positions). The MAKO robotic system had the highest number of publications in hip and knee surgery (*n* = 42), followed by Mazor in spine surgery (*n* = 41; Fig. 8). Six countries produced 14 types of surgical robots, with more than half originating from the United States (Fig. 9).

**Table 5:** Type of robotic orthopedic surgery and surgical site

Surgical site	Type of procedure	n (%)
Spine	Pedicle screw implantation	56 (89%)
	Vertebral augmentation	3 (5%)
	Laparoscopic anterior lumbar interbody fusion	1 (1%)
	Spine tumor resection surgery	1 (1%)
	Intraoperative localization	1 (1%)
	Anterior lumbar interbody fusion	1 (1%)
Knee	Total knee arthroplasty	24 (50%)
	Unicompartmental Knee Arthroplasty	23 (48%)
	Anterior cruciate ligament reconstruction	1 (2%)
Hip	Total hip arthroplasty	30 (100%)
Femur	Femoral neck cannulated screw placement	3 (60%)
	Intramedullary nail fixation	1 (20%)
	Core decompression of the femoral head	1 (20%)
Pelvis	Internal fixation of pelvic acetabular injuries	1 (25%)
	Percutaneous cannulated screw fixation,INFIX fixation, open reduction and internal plate fixation	1 (25%)
	Percutaneous screw placement combined with INFIX	1 (25%)
	Neurolysis	1 (25%)
Hand	Percutaneous internal fixation	1 (100%)
Elbow	Oberlin procedure	1 (100%)

### Study design

After excluding the article type of review, meta-analysis, case report, case series, and technique note, retrospective studies were involved in 56% (71/126) and prospective studies in 44% (55/126), respectively. The number of samples in different studies ranges from 10 to 1064, with 77% (97/126) of studies less than 100 sample sizes, 17% (21/126) between 100 to 300, and only 6% (8/126) with more than 300 sample sizes. From 152 clinical studies, 43% (65/152) were descriptive studies, followed by randomized controlled studies (21%, 32/152), case–control studies (20%, 30/152), and cohort studies (16%, 25/152).

### Hotspot and research trends

**Fig. 10:**
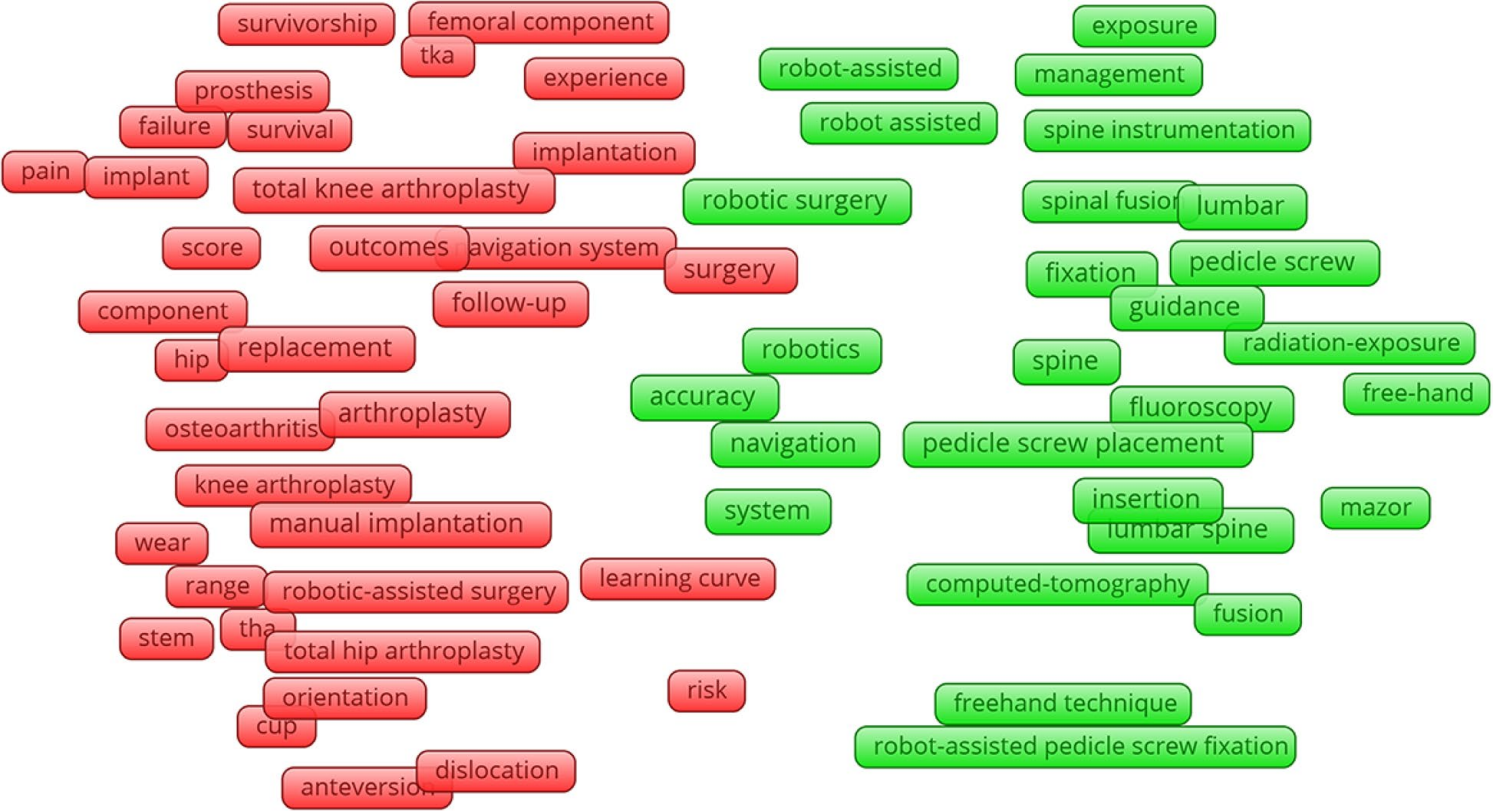
Co-occurrence network of robotic orthopedic surgery

**Fig. 11:**
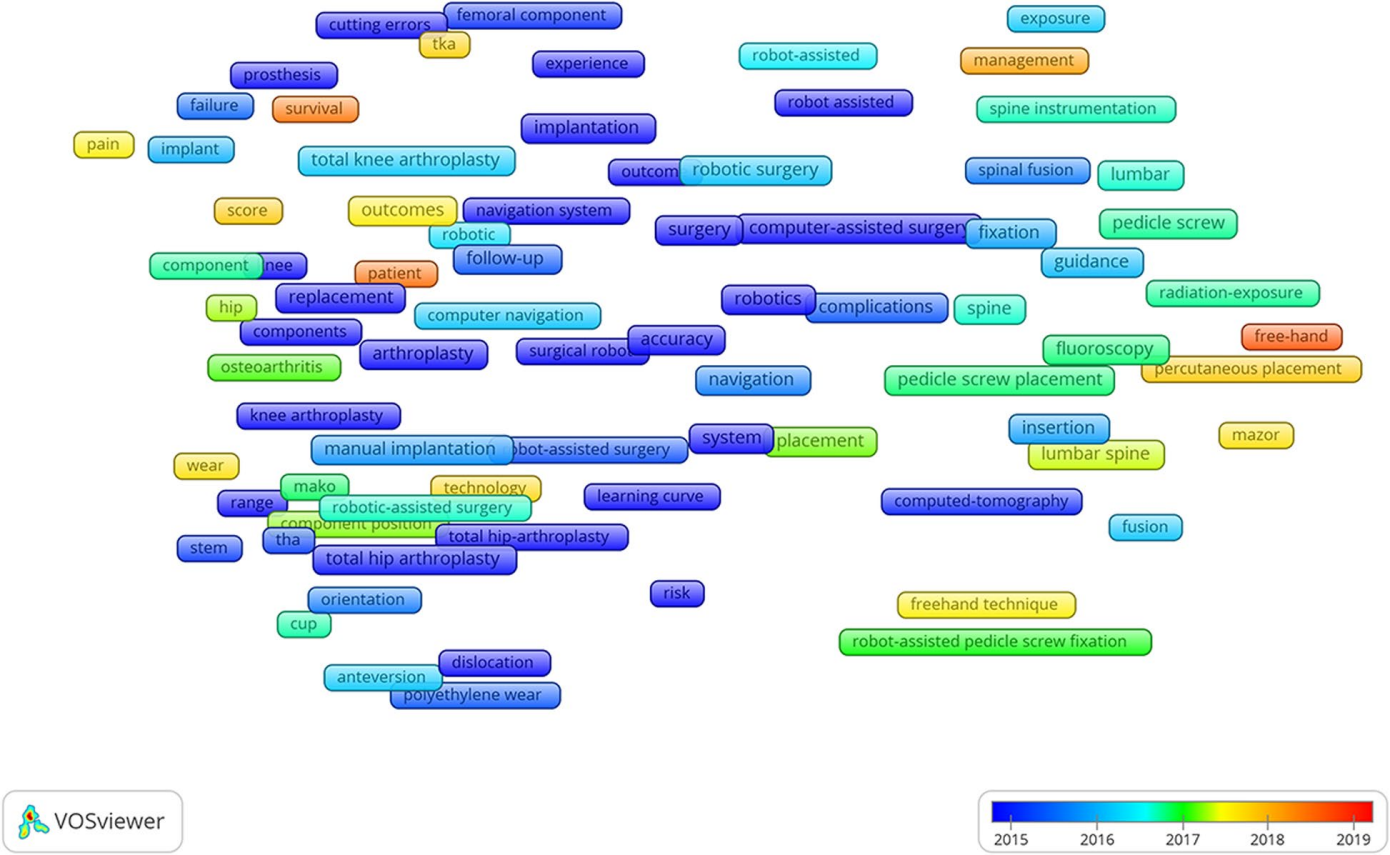
Overlay visualization from 2000 − 2019 in robotic orthopedic surgery

Nine hundred and fifty author keywords were exported from the included studies. The top 20 most frequently used keywords are presented in Table 6. All keywords (author keywords and keywords plus) were further analyzed by VOSviewer software, with the identification of two clusters from 112 keywords (occurrence number > 4). Co-occurrence networks represent different groups based on the frame color. The red-colored group was related to robotic surgery in joint replacement, with the most popular keywords “acetabular component”, “alignment”, and “anteversion”. The green-colored group was associated with robotics in spinal surgery, with the top three keywords “accuracy”, “complications”, and “computer navigation” (Fig. 10). The overlay visualization presents the occurrence time of keywords: the closer the color is to red, the closer the topic is to the present (Fig. 11). To observe changes in the research trends in these years, the keywords during 2000 − 2004, 2005 − 2009, 2010 − 2014, and 2015 − 2019 were exported separately and further analyzed by VOSviewer. The most number of keywords and new trends were between 2015 to 2019 (Additional file 2).

**Table 6:** Top 20 most frequently used keywords in robotic orthopedic surgery publications

Keywords	Number of occurrences
Robotics	43
Pedicle screw	33
Total knee arthroplasty	30
Unicompartmental knee arthroplasty	27
Navigation	25
Outcome	24
Spinal fusion	24
Robotic surgery	21
Minimally invasive	20
Total hip arthroplasty	17
Computer-assisted surgery	17
Accuracy	16
Arthroplasty	14
ROBODOC	9
MAKO	9
Learning curve	8
Complication	7
Freehand technique	7
TKA	7
Mazor	6

## Discussion

In the present study, we found 224 related papers from 2000 to 2019 using the WOS database. Bibliometric analysis was used to explore the characteristics of the robotic technique in orthopedics from multiple perspectives in this field, these findings most likely have implications for research and practice in future studies.

### Countries

Our bibliometric analysis reported an increasing trend in the number of global contributions from 2000 to 2019, with a marked rise since 2014. The United States had the greatest number of clinically relevant publications in robotic orthopedic surgery. From 2012, the United States ranked first as the country with the greatest number of publications for a consecutive eight years. Bibliographic coupling networks of countries showed three groups. The green group comprises mainly European countries, such as Germany, France, and Greece. The red group is distributed in countries from Asia, Europe, North America, and Oceania. The blue group comprised two Asian countries and Argentina. Based on the distance between countries, investigated topics between the Netherlands and Switzerland were similar, as well as between Germany and China. Research among the United States, the United Kingdom, and Israel was found to be comparable (Fig. 4). At present, only 23 countries are involved in orthopedic robot research. Wide popularization of the orthopedic robotic technique is still required in more countries to benefit patients.

### Authors and organizations

The authors Tian Wei and Mont Michael A were found to contribute most in the orthopedic robot field. The majority of the top nine authors with most contributions stemmed from the top three institutions with most contribution. With the exception of one institute in Germany, all other institutions with at least eight publications were from the United States, China, and the United Kingdom. Bibliographic coupling networks of institutions showed most institutions to be from the United States. The red group had the most number of institutions (*n* = 10) from 10 countries, the green group had 25 institutions from seven countries, and the blue group only seven institutions from the United States and Japan, respectively (Fig. 5). Among all institutions, research from the Cleveland Clinic, Beijing Jishuitan Hospital, and University of London was most relevant in orthopedic robotic research, originating from the United States, China, and the United Kingdom, respectively. These three countries occupy important positions in the clinical application of robotic orthopedic surgery.

### Citation and journals

Citations are an indicator that assesses the scholarly impact of publications. The present study showed the top 30 most cited papers in the clinical application of robotic orthopedic surgery. Scholars can easily and rapidly obtain the most influential articles on orthopedic robots in clinical use. By collecting journal information, we identified the journals interested in this area. From the number of publications by journals, 13 journals had at least six articles (Table 3), with the majority orthopedic specialty journals. The impact factor of the journal list showed only one journal with an impact factor greater than six (Table 4). In combination, these results indicate that most research was published in speciality journals, and there was an absence of recognition from general medical journals of high academic impact. Bibliographic coupling and co-citation network of the journal demonstrated that the *Journal of Arthroplasty* was the most relevant and influential journal in the orthopedic robot. The most evident characteristics were in the green circles, with most journals related to neurosurgery, orthopedic or joint surgery. This result may indicate that spinal robotic-related research is more concentrated in neurosurgery journals.

### Type of robotic surgery and location

From the type of robotic surgery and surgical site, orthopedic robotic products are focused mainly on spine and lower limb joints robots (Table 5). MAKO and Mazor was the most popular orthopedic robot in the lower limb joints and spine surgery (Fig. 8). The robot-assisted surgery of pedicle screw implantation and joint replacement have gradually matured, with these surgeries likely becoming the mainstream choice in spine and joint surgery in the future. Currently, only a few cases were performed on the femur, hand, and pelvis by fracture fixation, which was published in 2019 [27–30]. This phenomenon may suggest that the orthopedic robot will be further developed in trauma surgery. Robotic production technology is currently mastered in the hands of a limited number of countries, with most from the United States (Fig. 9). Due to lack of competition, it may create market monopolies and leads to retaining the high cost of the surgical robot. These high costs is a significant factor that can hinder the robotic technique development.

### Study design

Clinical trial related publications showed a greater proportion of retrospective, small sample populations and descriptive studies, with most of the current experience based on the low quality of research. More high-quality, large sample size, randomized controlled trials are still required.

### Hotspot and research trends

To determine research trends, we comprehensively analyzed the most widely used keywords and performed co-occurrence analysis. From the top 20 most frequently used keywords, in addition to keywords related to the surgical site, the terms “navigation”, “outcome”, “accuracy”, “learning curve”, “complication”, and “freehand technique” likely reflect the focus of robot orthopedic surgery [31–35]. Co-occurrence network results showed two research groups in this study, with red related to joint surgery, and green to spine surgery (Fig. 10). The results further supported those aforementioned from the type of surgery, with the orthopedic robot frequency used in joint and spinal surgery. Timeline visualization of co-occurrence showed an increasing trend in research on robotic orthopedic surgery, particularly from 2015 to 2019 (Additional file 2). Earlier studies concentrated on robotic techniques, gradually placing increasing focus on patient outcomes. In recent years, the surgeon appears more concerned with patient survival after robotic surgery, and the comparison between robotic surgery and freehand techniques [34, 36, 37].

## Limitations

The present study had several limitations. First, our bibliometric analysis was based on the WoS database, which potentially missed several publications from frequently used databases, such as Scopus and PubMed [38, 39]. Second, conference proceedings were excluded in the present study, as they could be published twice as a conference abstract and complete journal article [40, 41]. However, this may result in some potentially valuable information loss. Third, citation time is usually used to evaluate the quality of publications. We ranked the top 30 most cited papers to identify high academic impact publications in the clinical application of robotics in orthopedics. However, the number of citations was likely impacted by self-citation, time sequence of publications, and controversial articles. Fourth, we only performed the commonly used bibliographic coupling, co-citation analysis to find the relationship between institutions, journals, and countries. The visualization method of the co-authorship and citation analysis could also provide valuable information for bibliometrics [21, 42]. Some important information was most likely missed in the present study. Fifth, our study focused on the clinical use of robotics in orthopedics, with publications involving animals or cadaveric studies not considered.

## Conclusion

The use of robotic technology in clinical orthopedics is on the rise, with a sharp increase after 2014. Scholars and institutions of the United States, China, and the United Kingdom play an important role in this field. *The Journal of Arthroplasty* was the strongest correlation and academic impact journal in the field of orthopedic robotics. Most orthopedic robot research was published in orthopedic, spine, or joint surgery journals, and were absent in general medical journals of high academic impact, hence lacking recognition. Pedicle screw implantation and joint replacement are the current mainstream surgical procedures in orthopedic robots. Robotic technology in fracture fixation is promising for further development in trauma surgery. The majority of the quality of research was low, with future large-scale high-quality research required. Patient outcome and a comparison between robotic surgery and human techniques may become the next research trends.

## Supplementary Information

**Additional file 1.** Top 30 cited papers on robotic orthopedic surgery. The research hotspot of robotic orthopedic surgery during 2000 − 2004, 2005 − 2009, 2010 − 2014, and 2015 − 2019.

**Additional file 2.** Changes in research trends in robotic orthopedic surgery during 2000–2019.

## Data Availability

All data generated or analysed during this study are either included in this published article or its supplementary information files.

## Abbreviation

USA: United States of America
UK: United Kingdom
WoS: Web of Science
