# Supplementary material for: Clinical application of robotic orthopedic surgery: a bibliometric study
Source: BMC Musculoskelet Disord. 2021 Nov 22;22:968. doi: 10.1186/s12891-021-04714-7 (PMC8609816; doi:10.1186/s12891-021-04714-7)
Supplement: Supplementary file 1 — Additional file 1. Top 30 cited papers on robotic orthopedic surgery. The research hotspot of robotic orthopedic surgery during 2000 − 2004, 2005 − 2009, 2010 − 2014, and 2015 − 2019. [file 12891_2021_4714_MOESM1_ESM.zip › Additional file 1-.pdf]

Rank	Title	Journals	Citation counts
1	Hands-on robotic unicompartmental knee replacement - A prospective, randomised controlled study of the Acrobot system	JOURNAL OF BONE AND JOINT SURGERY-BRITISH VOLUME	153
2	Clinical Acceptance and Accuracy Assessment of Spinal Implants Guided With SpineAssist Surgical Robot Retrospective Study	SPINE	126
3	Perioperative course and accuracy of screw positioning in conventional, open robotic-guided and percutaneous robotic-guided, pedicle screw placement	EUROPEAN SPINE JOURNAL	119
4	Technique and first clinical results of robot-assisted total knee replacement	KNEE	106
5	Accuracy of Robot-Assisted Placement of Lumbar and Sacral Pedicle Screws A Prospective Randomized Comparison to Conventional Freehand Screw Implantation	SPINE	98
6	Comparison of robotic-assisted and manual implantation of a primary total hip replacement - A prospective study	JOURNAL OF BONE AND JOINT SURGERY-AMERICAN VOLUME	98
7	Percutaneous Placement of Pedicle Screws in the Lumbar Spine Using a Bone Mounted Miniature Robotic System First Experiences and Accuracy of Screw Placement	SPINE	87
8	Spinal Robotics: Current Applications and Future Perspectives	NEUROSURGERY	81
9	The hands-on orthopaedic robot "Acrobot": Early clinical trials of total knee replacement surgery	IEEE TRANSACTIONS ON ROBOTICS AND AUTOMATION	80
10	Robot-Assisted Unicompartmental Knee Arthroplasty	JOURNAL OF ARTHROPLASTY	79

11	Results of total hip replacement using the Robodoc surgical assistant system: clinical outcome and evaluation of complications for 97 procedures	INTERNATIONAL JOURNAL OF MEDICAL ROBOTICS AND COMPUTER ASSISTED SURGERY	72
12	Comparison of robotic-assisted and conventional manual implantation of a primary total knee arthroplasty	JOURNAL OF ARTHROPLASTY	68
13	Robotic systems in orthopaedic surgery	JOURNAL OF BONE AND JOINT SURGERY-BRITISH VOLUME	67
14	Comparison of Robotic-assisted and Conventional Acetabular Cup Placement in THA: A Matched-pair Controlled Study	CLINICAL ORTHOPAEDICS AND RELATED RESEARCH	65
15	Robotic-assisted TKA Reduces Postoperative Alignment Outliers and Improves Gap Balance Compared to Conventional TKA	CLINICAL ORTHOPAEDICS AND RELATED RESEARCH	64
16	Accuracy of Dynamic Tactile-Guided Unicompartmental Knee Arthroplasty	JOURNAL OF ARTHROPLASTY	63
17	Miniature robotic guidance for pedicle screw placement in posterior spinal fusion: early clinical experience with the SpineAssist (R)	INTERNATIONAL JOURNAL OF MEDICAL ROBOTICS AND COMPUTER ASSISTED SURGERY	62
18	Simultaneous bilateral total knee arthroplasty with robotic and conventional techniques: a prospective, randomized study	KNEE SURGERY SPORTS TRAUMATOLOGY ARTHROSCOPY	61
19	Robotics in Arthroplasty: A Comprehensive Review	JOURNAL OF ARTHROPLASTY	59
20	Safety and accuracy of robot-assisted versus fluoroscopy-guided pedicle screw insertion for degenerative diseases of the lumbar spine: a matched cohort comparison	JOURNAL OF NEUROSURGERY-SPINE	59
21	Robotic-assisted pedicle screw placement: lessons learned from	EUROPEAN SPINE JOURNAL	59

	the first 102 patients		
22	Robotic assisted spinal surgery - from concept to clinical practice	COMPUTER AIDED SURGERY	53
23	Improved Accuracy of Component Positioning with Robotic-Assisted Unicompartmental Knee Arthroplasty	JOURNAL OF BONE AND JOINT SURGERY-AMERICAN VOLUME	52
24	Clinical Pedicle Screw Accuracy and Deviation From Planning in Robot-Guided Spine Surgery	SPINE	50
25	Miniature robotic guidance for spine surgery - introduction of a novel system and analysis of challenges encountered during the clinical development phase at two spine centres	INTERNATIONAL JOURNAL OF MEDICAL ROBOTICS AND COMPUTER ASSISTED SURGERY	48
26	A Comparison between Robotic-assisted and Manual Implantation of Cementless Total Hip Arthroplasty	CLINICAL ORTHOPAEDICS AND RELATED RESEARCH	47
27	Robots in orthopaedic surgery - Past, present, and future	CLINICAL ORTHOPAEDICS AND RELATED RESEARCH	44
28	Minimally Invasive Robotic Versus Open Fluoroscopic-guided Spinal Instrumented Fusions A Randomized Controlled Trial	SPINE	42
29	Navigation and Robotics in Spinal Surgery: Where Are We Now?	NEUROSURGERY	41
30	Robot-assisted spine surgery: feasibility study through a prospective case-matched analysis	EUROPEAN SPINE JOURNAL	40
