# Supplementary material for: Clinical application of robotic orthopedic surgery: a bibliometric study
Source: BMC Musculoskelet Disord. 2021 Nov 22;22:968. doi: 10.1186/s12891-021-04714-7 (PMC8609816; doi:10.1186/s12891-021-04714-7)
Supplement: Supplementary file 2 — Additional file 2. Changes in research trends in robotic orthopedic surgery during 2000–2019. [file 12891_2021_4714_MOESM2_ESM.zip › Additional file 2..pdf]

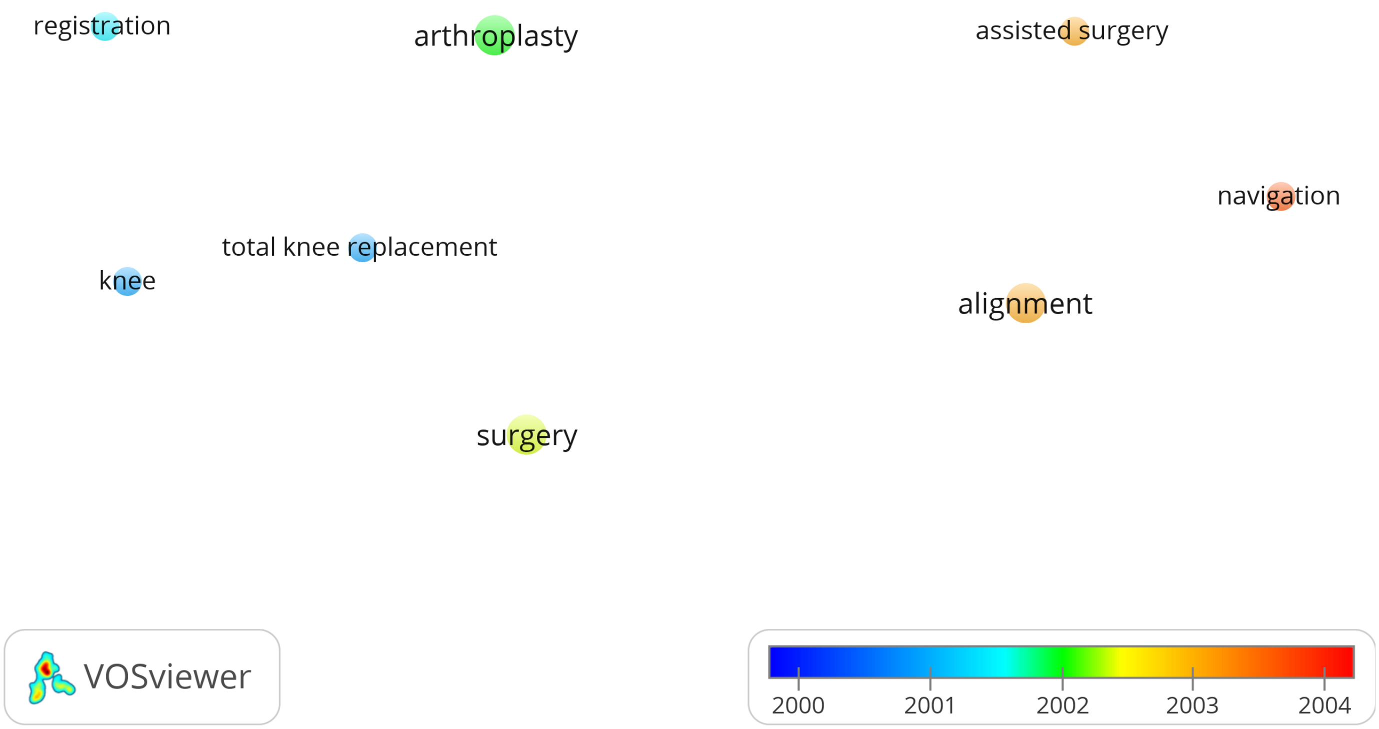
registration
arthroplasty
assisted surgery
navigation
total knee replacement
knee
alignment
surgery
VOSviewer
2000
2001
2002
2003
2004

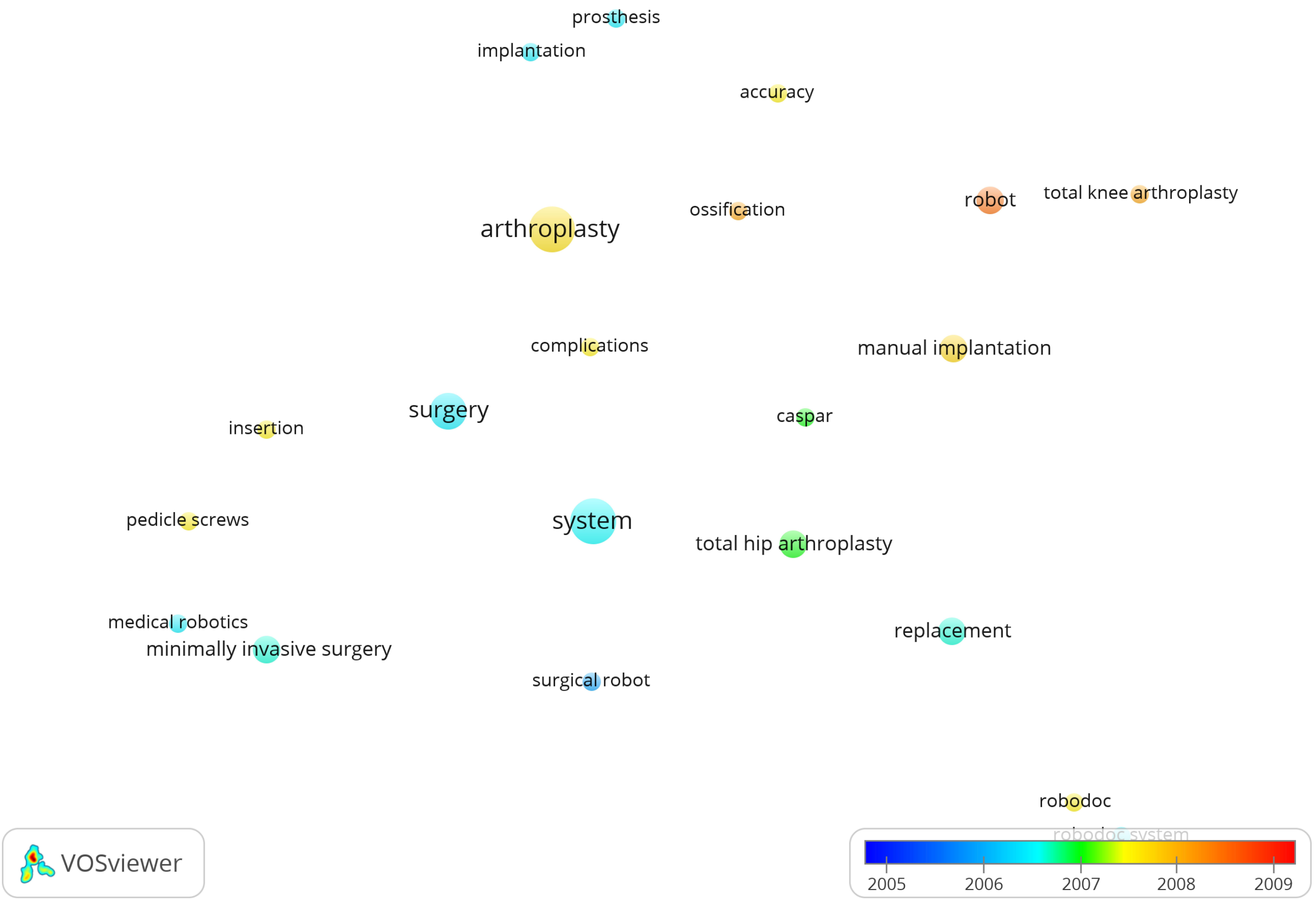

prosthesis
implantation
accuracy
arthroplasty
ossification
robot
total knee arthroplasty
complications
manual implantation
surgery
insertion
caspar
pedicle screws
system
total hip arthroplasty
medical robotics
minimally invasive surgery
surgical robot
replacement
robodoc
robodoc system
VOSviewer
2005
2006
2007
2008
2009

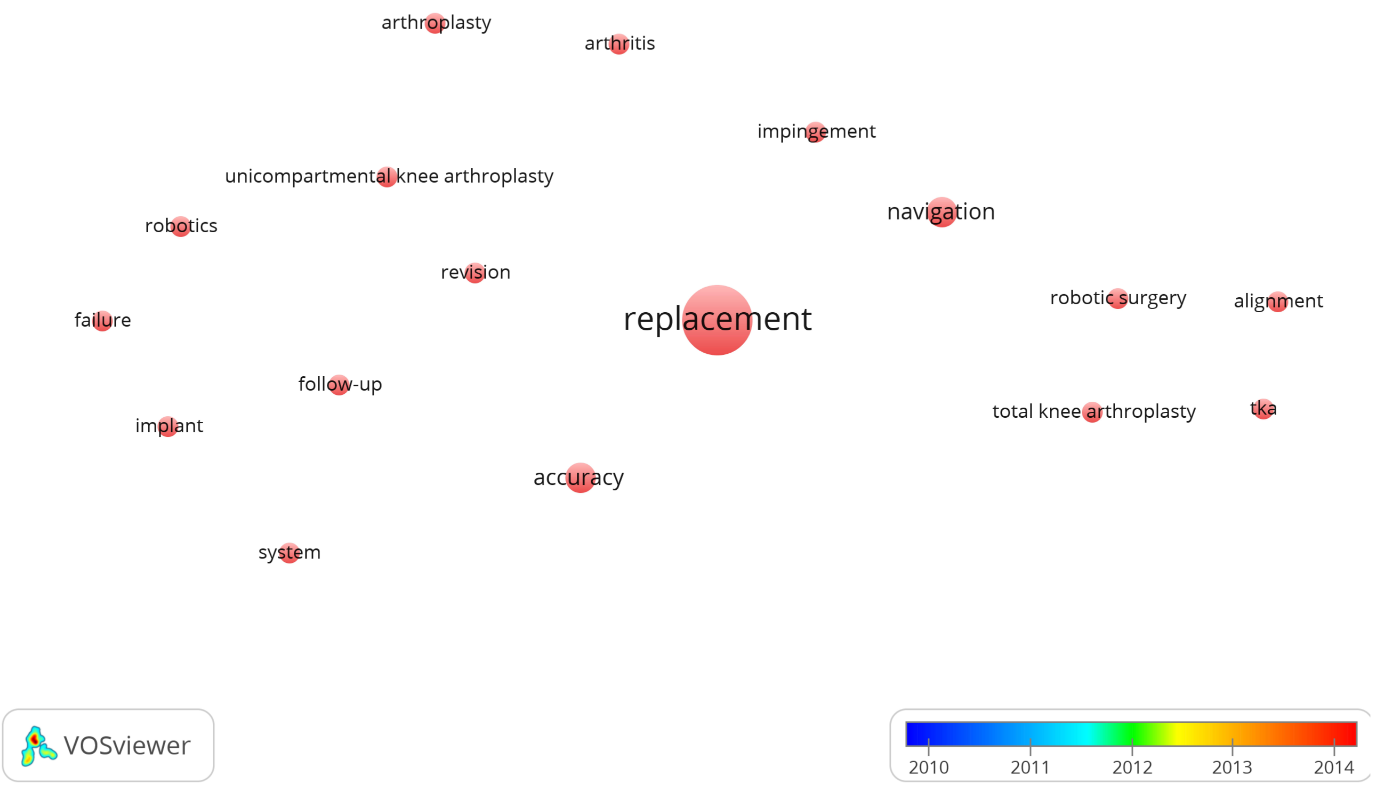
arthroplasty
arthritis
impingement
unicompartmental knee arthroplasty
navigation
robotics
revision
replacement
robotic surgery
alignment
failure
follow-up
total knee arthroplasty
tka
implant
accuracy
system
VOSviewer
2010
2011
2012
2013
2014

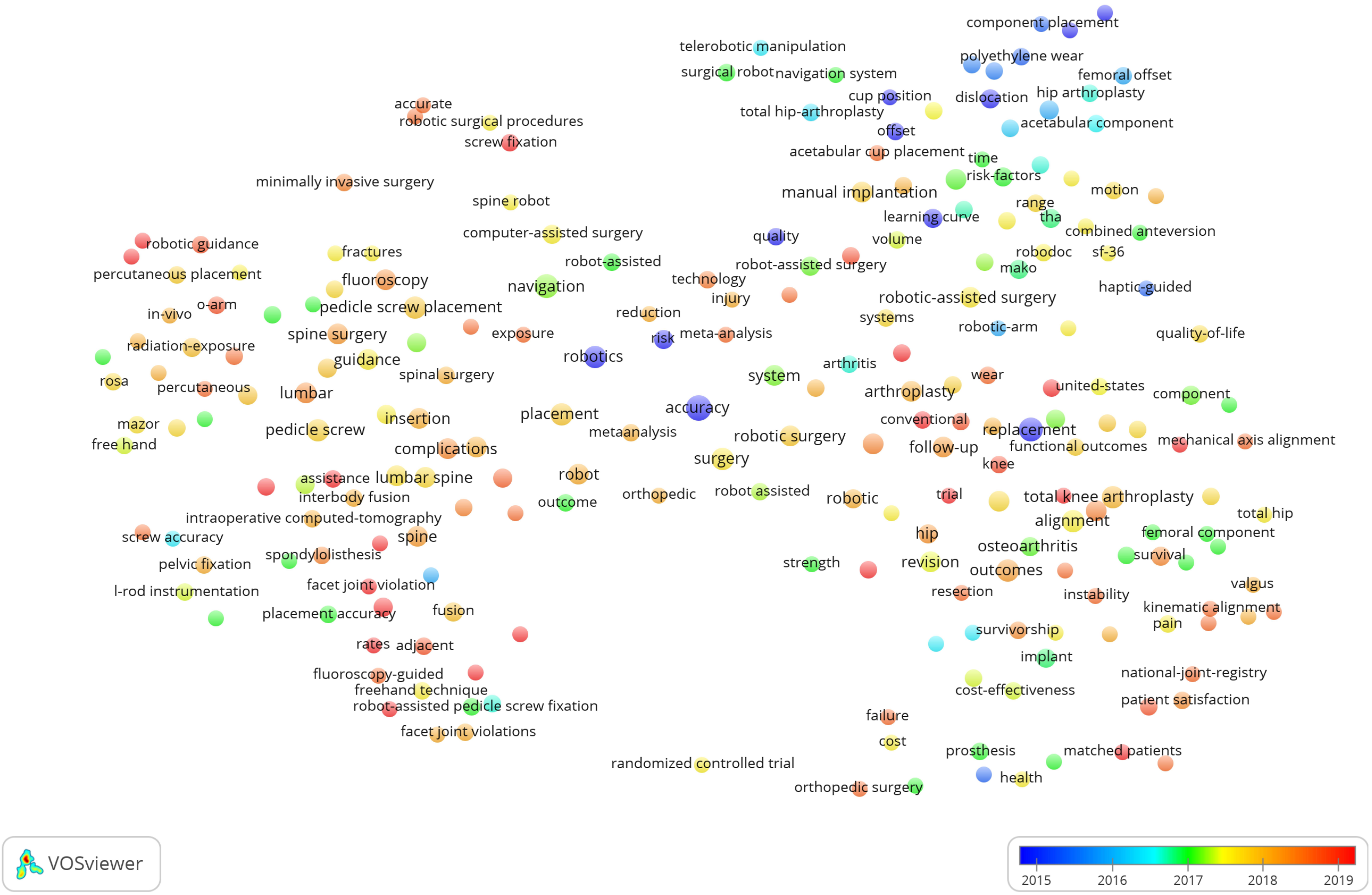
component placement
telerobotic manipulation
polyethylene wear
surgical robot
navigation system
femoral offset
accurate
cup position
dislocation
hip arthroplasty
robotic surgical procedures
total hip-arthroplasty
acetabular component
screw fixation
offset
acetabular cup placement
time
risk-factors
minimally invasive surgery
manual implantation
motion
spine robot
range
learning curve
tha
computer-assisted surgery
combined anteversion
quality
volume
robotic guidance
fractures
robodoc
sf-36
robot-assisted
robot-assisted surgery
mako
percutaneous placement
technology
fluoroscopy
navigation
haptic-guided
injury
robotic-assisted surgery
o-arm
in-vivo
pedicle screw placement
reduction
systems
robotic-arm
spine surgery
exposure
risk
meta-analysis
quality-of-life
radiation-exposure
guidance
robotics
arthritis
spinal surgery
rosa
system
wear
percutaneous
united-states
lumbar
arthroplasty
component
accuracy
mazor
insertion
placement
conventional
pedicle screw
replacement
metaanalysis
mechanical axis alignment
free hand
robotic surgery
follow-up
functional outcomes
complications
surgery
knee
assistance
lumbar spine
robot
orthopedic
robot assisted
trial
interbody fusion
outcome
robotic
total knee arthroplasty
total hip
intraoperative computed-tomography
alignment
screw accuracy
spine
hip
femoral component
osteoarthritis
spondylolisthesis
strength
revision
survival
pelvic fixation
outcomes
valgus
facet joint violation
resection
instability
l-rod instrumentation
kinematic alignment
placement accuracy
fusion
pain
survivorship
rates
adjacent
implant
fluoroscopy-guided
national-joint-registry
freehand technique
cost-effectiveness
robot-assisted pedicle screw fixation
patient satisfaction
failure
facet joint violations
cost
prosthesis
matched patients
randomized controlled trial
health
orthopedic surgery
VOSviewer
2015
2016
2017
2018
2019
