# Supplementary material for: Clinical application of robotic orthopedic surgery: a bibliometric study
Source: BMC Musculoskelet Disord. 2021 Nov 22;22:968. doi: 10.1186/s12891-021-04714-7 (PMC8609816; doi:10.1186/s12891-021-04714-7)
Supplement: Supplementary file 1 — Additional file 1. Top 30 cited papers on robotic orthopedic surgery. The research hotspot of robotic orthopedic surgery during 2000 − 2004, 2005 − 2009, 2010 − 2014, and 2015 − 2019. [file 12891_2021_4714_MOESM1_ESM.zip › Legend for Additional file1.doc]

Additional file 1-Top 30 cited papers on robotic orthopedic surgery. The research hotspot of robotic orthopedic surgery during 2000−2004, 2005−2009, 2010−2014, and 2015−2019.
