# Supplementary material for: Clinical application of robotic orthopedic surgery: a bibliometric study
Source: BMC Musculoskelet Disord. 2021 Nov 22;22:968. doi: 10.1186/s12891-021-04714-7 (PMC8609816; doi:10.1186/s12891-021-04714-7)
Supplement: Supplementary file 2 — Additional file 2. Changes in research trends in robotic orthopedic surgery during 2000–2019. [file 12891_2021_4714_MOESM2_ESM.zip › Legend for Additional file2.doc]

Additional file 2-Changes in research trends in robotic orthopedic surgery during 2000–2019.
